# Single and interactive effects of variables associated with climate change on wheat metabolome

**DOI:** 10.3389/fpls.2022.1002561

**Published:** 2022-10-10

**Authors:** Kristýna Večeřová, Michal Oravec, Swati Puranik, Hana Findurová, Barbora Veselá, Emmanuel Opoku, Kojo Kwakye Ofori-Amanfo, Karel Klem, Otmar Urban, Pranav Pankaj Sahu

**Affiliations:** ^1^ Laboratory of Ecological Plant Physiology, Global Change Research Institute of the Czech Academy of Sciences, Brno, Czechia; ^2^ Department of Agrosystems and Bioclimatology, Faculty of AgriSciences, Mendel University in Brno, Brno, Czechia; ^3^ Department of Forest Ecology, Faculty of Forestry and Wood Technology, Mendel University in Brno, Brno, Czechia

**Keywords:** climate change, wheat, metabolomics, physiology, elevated CO_2_, temperature, drought

## Abstract

One of the key challenges linked with future food and nutritional security is to evaluate the interactive effect of climate variables on plants’ growth, fitness, and yield parameters. These interactions may lead to unique shifts in the morphological, physiological, gene expression, or metabolite accumulation patterns, leading to an adaptation response that is specific to future climate scenarios. To understand such changes, we exposed spring wheat to 7 regimes (3 single and 4 combined climate treatments) composed of elevated temperature, the enhanced concentration of CO_2_, and progressive drought stress corresponding to the predicted climate of the year 2100. The physiological and metabolic responses were then compared with the current climate represented by the year 2020. We found that the elevated CO_2_ (eC) mitigated some of the effects of elevated temperature (eT) on physiological performance and metabolism. The metabolite profiling of leaves revealed 44 key metabolites, including saccharides, amino acids, and phenolics, accumulating contrastingly under individual regimes. These metabolites belong to the central metabolic pathways that are essential for cellular energy, production of biosynthetic pathways precursors, and oxidative balance. The interaction of eC alleviated the negative effect of eT possibly by maintaining the rate of carbon fixation and accumulation of key metabolites and intermediates linked with the Krebs cycle and synthesis of phenolics. Our study for the first time revealed the influence of a specific climate factor on the accumulation of metabolic compounds in wheat. The current work could assist in the understanding and development of climate resilient wheat by utilizing the identified metabolites as breeding targets for food and nutritional security.

## Introduction

Plants are sensitive to changes in temperature, water availability, and atmospheric CO_2_ concentration (Thornton et al., 2014). As reported by the Intergovernmental Panel on Climate Change (IPCC), extreme climate events like heat waves and drought periods will become more intense and frequent, which could have devastating consequences on plant growth, development, and production (IPCC, 2018). The combinations of these climate factors may have even a more severe impact on food availability to the increasing world population. The strategy to improve the resilience of economically important crops has mostly relied on the screening of genotypes by exposing them to a single climate factor (e.g., drought, temperature, CO_2_, etc.). However, the adaptive response under simultaneous exposure to multiple climate factors may vary widely, resulting in an undesired or failed crop selection process. Therefore, to prevent such crop failure in future, an assessment of the impact of combined treatments on plant’s performance and adaptability is required.

Plants respond to stress by adjusting their morpho-physiological status such as by inhibiting photosynthesis (Jagtap et al., 1998), and/or altering vegetative growth and biomass allocation (Pandey et al., 2015). The molecular mechanisms of acclimation constitute the activation of genes for adaptive responses, such as the heat shock proteins (HSPs), and/or enzymes and additional transcripts facilitating detoxification and signaling of reactive oxygen species (ROS) (Rizhsky et al., 2004). In addition, under severe conditions, membrane, and protein damage lead to the accumulation of lipid peroxidation products (Jiang and Huang, 2001). This induces altered redox homeostasis and increase in ROS and consequently enhanced cellular oxidative damage (Krasensky and Jonak, 2012; Munné-Bosch et al., 2012). In addition, a wide range of different metabolites such as primary metabolites (carbohydrates, tricarboxylic acid cycle intermediates, and amino acids) essential for the growth and development, secondary metabolites (phenolics, terpenes, and nitrogen-containing compounds) along with the hormones (such as abscisic acid, jasmonic acid, salicylic acid, and ethylene) are also produced to regulate plant climate response (Fang et al., 2019; Kumar et al., 2020; Kumari et al., 2020). The primary metabolites, such as amino acids, play a pivotal role in enhancing fitness under various environmental cues. For example, the accumulation of amino acid proline is considered an indicator of adaptive behavior of plants exposed to various abiotic factors (Hayat et al., 2012; Fàbregas and Fernie, 2019; Girija et al., 2022). It helps in the maintenance of cell turgidity and reduction in electrolyte leakage to prevent cellular oxidative bursts (Hayat et al., 2012). Besides being the major metabolic resource and structural constituent of cells, carbohydrates such as sucrose and hexoses, also regulate stress-signaling and control the expression of growth as well as stress-related genes (Rosa et al., 2009). The TCA cycle intermediates are also involved in providing environmental stress tolerance attributes (Tahjib-Ul-Arif et al., 2021) and their accumulation under drought and heat stress has been reported to assist in the maintenance of physiological performance by regulating the photosynthetic rates and cellular redox status (Fernie et al., 2004; Das et al., 2017). On the other hand, secondary metabolites mitigate the plant’s survival by protecting it from adverse environments (Agostini-Costa et al., 2012; Kurepin et al., 2017). For example, the antioxidants nature of flavonoids (a major class of phenylpropanoids) regulates various functions such as maintaining water homeostasis (Naing and Kim, 2021), and stomatal dynamics (Li et al., 2021) to improve the crop stress adaptability.

Identification of changes in the composition of such key compounds and derivate through the metabolomic approach serves as a powerful strategy to advance our understanding of the adaptation responses in plants (Obata and Fernie, 2012; Sardans et al., 2020; Girija et al., 2022). Various studies have provided novel insights into the metabolic signature among crop species (e.g., rice, maize, and wheat) wide-ranging across growth stages and genotypes (Peng et al., 2017; Xu et al., 2019; Chen et al., 2020). However, investigating the effects of climate change is complex because multiple interactions between various environmental factors could prompt unique shifts in the biochemical composition. A recent study in Arabidopsis revealed the difference in the metabolite accumulations under single and multi-climate factors (Zinta et al., 2018). While elevated CO_2_ was found to largely enhance the cellular concentration of saccharides and amino acids, the combination with other factors caused the opposite effect on the accumulation of these compounds. Very few studies have been done to identify the shifts in metabolite pattern under combined heat and drought stress, particularly in crops (Obata et al., 2015; Templer et al., 2017). These studies revealed that most of the metabolic changes are additive, i.e., represent the sum of responses to the individual factors, and drought contributed most to such changes.

Bread wheat (*Triticum aestivum*) is a major cereal crop and widely cultivated for its grains (Shewry, 2009). The climate variability risks the livelihood and food security of about ~2 billion people who rely on wheat globally. The decline in the climate resilience of most of the wheat cultivars in the last 15 years was already observed in Europe, due to the repeated selection for few desirable traits (Kahiluoto et al., 2019). As most traits are complex and multigenic in nature, impact assessment of environmental stress on wheat crop relies not only on the type of factor exposed but also on its phenological growth stage. For example, the water availability at tillering is a critical phase in terms of grain yield potential (Curtis et al., 2002). Depending upon the severity and duration of drought stress, flowering and grain-filling phases are also considered as key stages which may result in the substantial yield loss (Farooq et al., 2014). The flowering stage is more sensitive to temperature stress which regulates the seed-setting and grain quality (Aiqing et al., 2018). The sensitivity of wheat to any of these climate variables can result in altered metabolic processes coupled with lower biomass accumulation and grain yield (Ihsan et al., 2016). Despite the advancement in knowledge, the impact of climate factor combinations on metabolite accumulation in wheat remains ambiguous. Therefore, considering its economic importance, we aimed to examine its acclimation responses to current as well as future climate scenarios by using an experimental model of single and combined climate treatments. This work allowed us to identify the most important compounds contributing to metabolome variations in wheat leaves under multifactorial treatments and their potential to the risks associated with climate change. The key metabolites and pathways identified in this study could be the potential targets to understand their future climate-induced regulation, and development of nutritionally enriched crops.

## Materials and methods

### Experimental regimes and plant growth set-up

The experiment started with setting climate conditions representing the typical growth environment of Central Europe for spring wheat. Three major climate variables representing key factors associated with an ongoing climate change, i.e., air temperature, water availability and atmospheric CO_2_ concentration were used. The data for air temperature (daily maximum, minimum and average) representing the years 2020 (current) and 2100 (future) were retrieved from the World Bank portal (https://climateknowledgeportal.worldbank.org/country/czech-republic/climate-data-projections). For the control set-up, an hourly dynamic of temperature (ambient temperature; aT; maximum/minimum= 24/10°C), constant CO_2_ concentration (ambient CO_2_; aC; 400 ± 50 ppm) and well-watered soil (water holding capacity; WHC = 90 ± 5%) conditions were maintained to mimic the environment for the year 2020. To understand the influence of individual climate variables and their combinations on acclimation responses in the year 2100, seven experimental setups were used. These included single factor treatments comprising of elevated temperature (eT), elevated CO_2_ (eC) and drought (D), and combinations of two-factors (eT+D, eC+D, eC+eT) and three-factors (eC+eT+D). For these setups, the chambers were enabled with the daily air temperature of maximum/minimum= 28/12°C (eT) and CO_2_ concentration of 700 ppm (eC). The applied 700 ppm CO_2_ concentration as an elevated condition was based on the projections for year 2100 (Meehl et al., 2007) as well as previous studies (Reyenga et al., 2001; Jauregui et al., 2015; Soba et al., 2019). Further, a progressive drought was applied by with-holding the water supply after 7 days of optimization until WHC reached 25 ± 5% and kept constant thereafter throughout the experiment. As each setup had different evaporation rates, a minimum of 10-days continuous drought was applied depending upon the onset of 25% WHC. The pattern of daily changes in the relative humidity (RH; maximum day/night, 65/90%), maximum day light intensity (800 µmol/m^2^/s) and light duration (15/9 h) were kept as constant for all experimental set-ups. The selection of light intensity (800 µmol/m^2^/s) was based on previous studies conducted on wheat to study the impact of single or multiple climate factors in a growth cabinet conditions (Nuttall et al., 2018; Chavan et al., 2019).

The spring wheat cultivar Cadenza was selected for our study as it the most widely used research variety (Fernández-Gómez et al., 2020) and genomic resources are also available for further study (Walkowiak et al., 2020). Seeds after stratification (4°C/48 h) and germination (24°C/48 h) were planted in the standard substrate (TS2; Klasmann-Deilmann, Geeste, Germany). Pots (dimension: 11×11×25 cm) containing the seedlings were later transferred to climate chambers (FytoScope FS-SI 3400; PSI, Drásov, Czech Republic) for respective treatments. Seedlings were initially allowed to optimize for seven days under temperature (daily maximum/minimum= 15/10°C), constant CO_2_ concentration (400 ± 50 ppm), RH (daily minimum/maximum, 65/90%), maximum/minimum light intensity (800/0 µmol/m^2^/s), light duration (15/9 h) and water saturated conditions. Afterwards, they were subjected to the control and treatment/experimental set-ups (detailed above) with each setup containing at least three plants per treatment (n=3). All physiological measurements and sampling for metabolite profiling were done on the youngest fully developed leaves (second from the top) of the primary shoot at end of the tillering stage (Feekes-6) as it is an agronomically important developmental stage that contributes to grain productivity (Khadka et al., 2020). All samplings and measurements were conducted between 11.00 and 14.00 hours Central European Time (CET), when the temperature and light intensity reached maxima and relative air humidity reached a minimum for the day.

### Physiological measurements

The measurements of photosynthesis and photosynthesis-related processes were done using Li-6800 gas-exchange system (LI-COR Biosciences, Lincoln, Nebraska, USA). To determine light-saturated CO_2_ assimilation rate (*A*), stomatal conductance (*Gsw*), transpiration rate (*Tr*), and water use efficiency (WUE = *A*/*Tr*) the leaves investigated were exposed to the light intensity of 1200 µmol/m^2^/s, while other microclimatic conditions inside the assimilation chamber were kept constant corresponding to daily maxima of individual treatments: CO_2_ concentration (400 ppm or 700 ppm for *aC* and eC regimes, respectively), leaf temperature (24°C or 28°C) and VPD (1.04 kPa and 1.32 kPa for aT and eT regimes, respectively). The measurements were conducted on three biological replicates. All data after testing for normal distribution were subject to one- or two-way analysis of variance (ANOVA) followed by *post-hoc* comparison using Tukey’s multiple range test (*P ≤ 0.05*). Principal component analysis (PCA) was used to analyze relations between variables. All statistical analyses were performed using the STATISTICA package version 12 (Stat Soft, Tulsa, Oklahoma, USA).

### Metabolite profiling of wheat leaf at vegetative stage

The metabolomic analysis was performed using the established protocol for the metabolic studies in cereals in the laboratory of metabolomics and isotopic analyses at the CzechGlobe (Klem et al., 2019). Briefly, detached leaves at 25-days post-treatments were immediately stored in liquid nitrogen. Lyophilized samples (100 mg dry weight) were subsequently homogenized and extracted using a methanol: chloroform: H_2_O solution (1:2:2). Aliquots of the polar phase were used to analyze saccharides, phenolic compounds, amino acids, phytohormones, and Krebs cycle intermediates employing an UltiMate 3000 system of high-performance liquid chromatography (HPLC) coupled with an LTQ Orbitrap XL high-resolution mass spectrometer (HRMS) (ThermoFisher Scientific, Waltham, Massachusetts, USA). The HRMS is equipped with a heated electrospray ionization source and was operated in full scan mode with a resolution of 60000. For the separation of individual compounds, a Hypersil GOLD chromatographic column (ThermoFisher Scientific, Waltham, Massachusetts, USA) of 150 mm (length), 2.1 mm (ID), and 3 μm (film thickness) was used. Moreover, gas chromatography coupled with mass spectrometry (GC-MS) was employed to analyze a spectrum of fatty acids, sterols, saccharides, and other non-polar compounds. Analyses were performed with a TSQ Quantum XLS triple Quadrupole (ThermoFisher Scientific, Waltham, Massachusetts, USA) on a Rxi-5Sil MS capillary column (Restek, Bellefonte, Pennsylvania, USA) of 30 m (length) with 5 m of integra-Guard, 0.25 mm (ID) and 0.25 µm (film thickness). The compounds detected by the chromatography were identified based on the comparison with the own mass library which had been created from the measurement of standards using HPLC-HRMS and GC-MS in full scan mode and containing about 300 compounds. The statistical analysis such as partial least squares discriminant analysis (PLS-DA), mean decrease accuracy (MDA), one-way ANOVA of the identified metabolites was done using MetaboAnalyst software (Pang et al., 2021). Correlation analyses were performed using the STATISTICA package version 12 (Stat Soft, Tulsa, Oklahoma, USA).

## Results

### Setting the regimes to study the impact of climate

**Figure 1:**
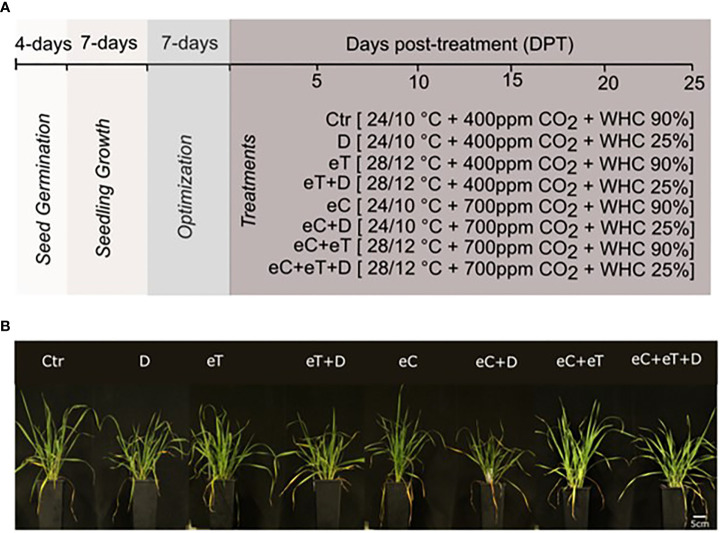
A schematic representation of experimental pipeline and developmental changes monitored in wheat cv. Cadenza. **(A)** Climate setup. Experimental setup to mimic the natural daily temperature dynamics (Tmax/Tmin) in June of years 2020 and 2100, atmospheric CO_2_ concentration [ambient (400 ppm) and elevated (700 ppm)] and water availability [water holding capacity (WHC) at 25 ± 5% (Drought, D) and 90 ± 5% (water saturated pots)]. The symbol of ± indicates fluctuations in the parameters. **(B)** Phenotypic differences observed at 25 DPT showing the variable effects of treatments. The bar scale represents 5 cm. Ctr, control treatment; eC, elevated CO_2_; eT, elevated temperature; D, drought; eC+eT, elevated CO_2_ and elevated temperature; eC+D, elevated CO_2_ and drought; eT+D, elevated temperature and drought; eC+eT+D, elevated CO_2_, elevated temperature, and drought.

As predicted by RCP 8.5 scenarios of IPCC, temperature, drought, and atmospheric CO_2_ concentration were used as the three major factors to study the impact of climate change (**Figure 1A**). Current (2020) and expected future (2100) growing conditions in Central Europe corresponding to June were simulated as described in the materials and methods. The growth of plants was monitored regularly until 25 day-post treatment (DPT). The tiller and leaf number were counted after 25 DPT, however, there was no significant difference at this stage (**Figure 1B**).

### Concentration of CO_2_ determines the physiological responses

**Figure 2:**
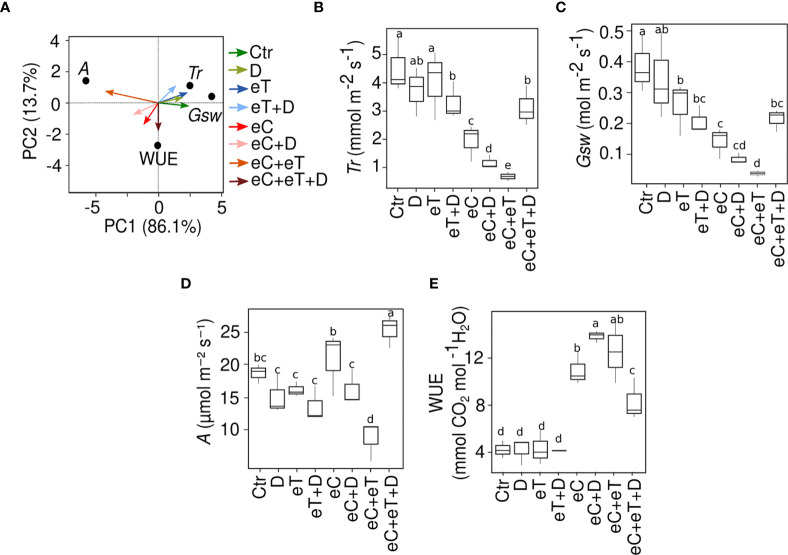
Impact of single and multiple climate variables on the physiology of wheat cv. Cadenza in tillering stage. **(A)** Principal component analysis plot of the physiological traits measured at 25 DPT. An account of **(B)** Transpiration rate, *Tr*; **(C)** Stomatal conductance, *Gsw*; **(D)** Rate of photosynthesis, *A*; **(E)** Water use efficiency, WUE, under single and combined climate variable treatments. Three biological replicates (*n*=3) per treatment were used to evaluate the physiological traits. The box plot shows the inter-quartile range with the mean. Values marked with the same letter do not differ according to Tukey’s test multiple range tests (*P* ≤ 0.05). Ctr, control treatment; eC, elevated CO_2_; eT, elevated temperature; D, drought; eC+eT, elevated CO_2_ and elevated temperature; eC+D, elevated CO_2_, and drought; eT+D, elevated temperature, and drought; eC+eT+D, elevated CO_2_, elevated temperature, and drought.

It was revealed that the pattern of change in the physiological parameters was mostly treatment-specific (**Figures 2A** **–E**). The variance (PC1 ~86%) was clearly observable due to the effect of eT (**Figure 2A**). Transpiration rate (*Tr*) and stomatal conductance (*Gsw*) significantly declined (*P* ≤ 0.05) under eC, eC+eT and eC+D treatments in comparison to the control condition (**Figures 2B, C**). There was no clear trend in the pattern of the photosynthesis rate (*A*) except for an increase under eC and eC+eT+D conditions (**Figure 2D**). On the contrary, eC+eT treatment led to a significant reduction of *A* values. Noticeably, eC alone or in combinations with other factors significantly (*P* ≤ 0.05) increased the water use efficiency (WUE), although this effect was less pronounced under eC+eT+D condition. These findings indicate a critical role of CO_2_ concentration in the adjusting of physiological state under future conditions.

### Metabolites accumulate differentially under various regimes

**Figure 3:**
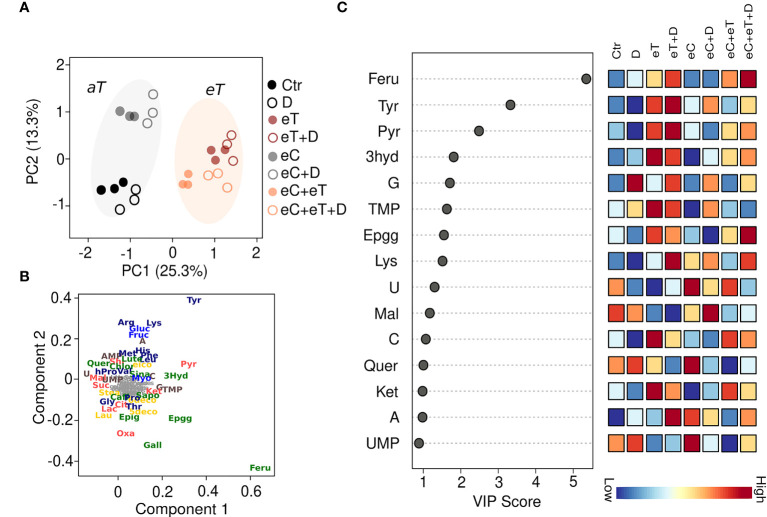
A supervised Partial Least Squares Discriminant Analysis (PLS-DA) of all the metabolites accumulated in the leaves of wheat cv. Cadenza after exposure to climate regimes. **(A)** Scores plot showing the distribution of metabolites based on the regimes. PC1 separated the metabolites based on the ambient (*aT*, gray circle) and elevated temperature (*eT*, orange circle). **(B)** Loading plot showing the metabolites discriminated based on PLS-DA. The metabolites such as phenolics (green), amino acids (dark blue), Krebs cycle intermediates (red), lipids (yellow) and nitrogenous bases (brown) have been shown. **(C)** The metabolites ranked by variable importance in projection (VIP). Ctr, control treatment; eC, elevated CO_2_; eT, elevated temperature; D, drought; eC+eT, elevated CO_2_ and elevated temperature; eC+ D, elevated CO_2_ and drought; eT+D, elevated temperature and drought; eC+eT+D, elevated CO_2_, elevated temperature and drought. The color code represents the specific metabolite families: dark blue, amino acid; light blue, saccharides; green, phenolics; yellow, fatty acids; red, Kreb’s cycle acids; brown, nucleotides. Compounds in grey represent non-significant (*P* ≤ 0.05) metabolites among the treatments identified by one-way ANOVA. Three biological replicates (*n*=3) per treatment were used in the study.

Overall, 77 metabolites were identified by GC-MS and HPLC-HRMS analysis among the leaf samples exposed to different climate regimes (**Supplementary Table 1**). Out of these, 44 metabolites significantly (*P* ≤ 0.05) varied among the treatments (**Supplementary Table 2**). The most influenced metabolite groups included amino acids (16), Krebs cycle intermediates (8), phenolics (10), saccharides (4), fatty acids (3) and nitrogenous bases (3) (**Supplementary Table 2**). A distinct separation of metabolites between the regimes with *aT* and those with *eT* was observed, accounting for ~20% of the variation (PC1; **Figure 3A**). This finding indicates that temperature has a strong effect on the metabolome. The main reasons for the metabolite differences in different regimes were identified by Partial least square discriminant analysis (PLS-DA). Based on their VIP scores, which is the measure of a variable’s importance, the metabolites such as tyrosin (Tyr), ferulic acid (Feru), pyruvic acid (Pyru), 3-hydroxybenzoic acid (3hyd), epigallocatechin gallate (Epgg), guanine (G), uracil (U), and thymidine monophosphate (TMP) showed largest differences among all the regimes (**Figure 3B**). Metabolites having VIP score >1.5 were considered significant (**Figure 3C**). The accumulation patterns of individual metabolite categories under the subjected regimes are described in detail below. Overall, the results show a treatment-specific accumulation of metabolites during the vegetative growth stage in spring wheat.

**Figure 4:**
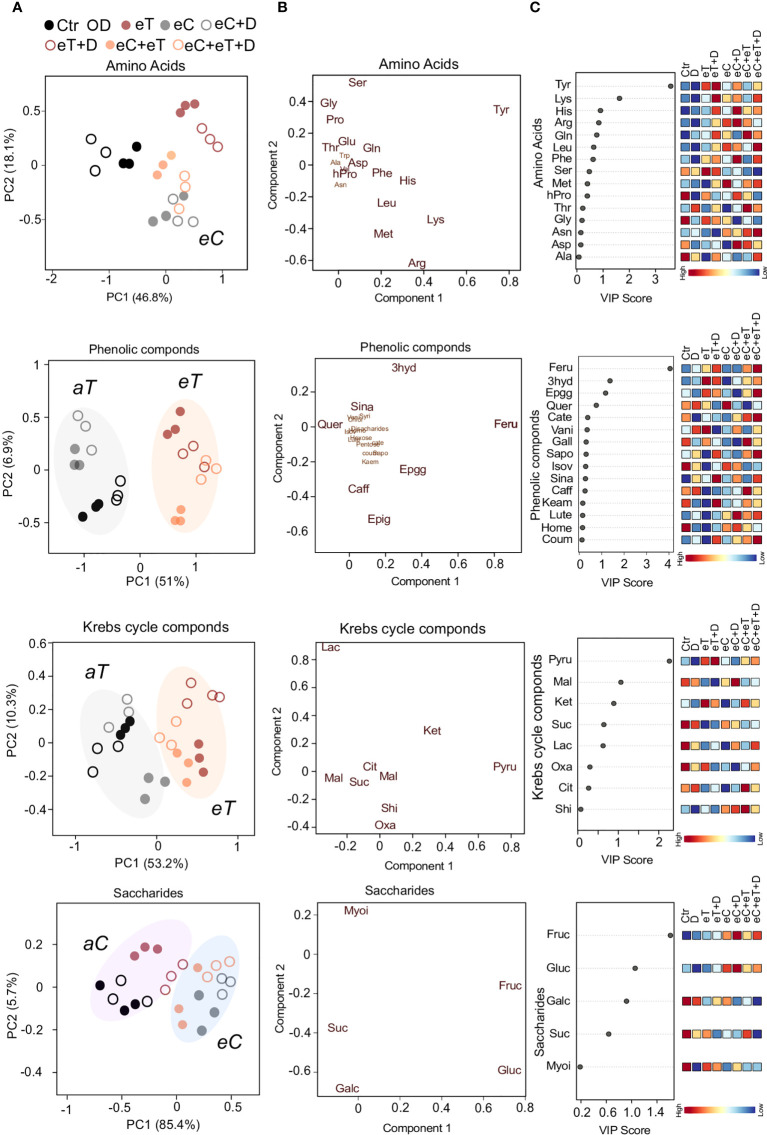
A supervised Partial Least Squares Discriminant Analysis (PLS-DA) of key metabolite categories accumulated in the leaves of wheat cv. Cadenza after exposure to climate regimes. **(A)** Scores plot showing the distribution of metabolites (amino acids; phenolics, Krebs cycle intermediates; saccharides) based on the regimes. Component 1 separated the metabolites based on the ambient temperature (aT, gray circle), elevated temperature (*eT*, orange circle). Ambient CO_2_ (*aC*, purple circle), and elevated CO_2_ concentrations (eC, blue circle). **(B)** Loading plot showing the metabolites (amino acids; phenolics, Krebs cycle intermediates; saccharides) discriminated based on PLS-DA. The metabolites in the bigger font significantly (*P* ≤ 0.05) varied among the treatments. **(C)** The metabolites ranked by variable importance in projection (VIP). Scale bar represents high and low VIP scores corresponding to the metabolites. Ctr, control treatment; eC, elevated CO_2_; eT, elevated temperature; D, drought; eC+eT, elevated CO_2_ and elevated temperature; eC+ D, elevated CO_2_ and drought; eT+D, elevated temperature and drought; eC+eT+D, elevated CO_2_, elevated temperature and drought. Abbreviations of the metabolites are provided in **Supplementary Table 1**.

Out of 19 amino acids detected in the metabolite profiling, 16 amino acids were significantly (*P ≤ 0.05*) accumulated among the treatments (**Supplementary Table 2**). These included branched chain amino acids (BCAAs) such as Leucine (Leu), aromatic (Tryptophan; Try, Phenylalanine; Phe, and Tyrosine; Tyr), hydrophilic-basic (Lysine; Lys, Arginine; Arg and Histidine; His) and hydrophobic (Glycine; Gly, Proline; Pro, Hydroxy-proline; Hpro, Methionine; Met) amino acids (**Supplementary Table 2**). The first component (46.8%) separated the *eT* and *eC* regimes from other treatments (**Figure 4A**). Further, PLS-DA analysis discriminated Tyr and hydrophilic-basic amino acids from the group of all amino acids (**Figure 4B**). The VIP score (>1.5; *P ≤ 0.05*) suggested Tyr and Lys as the key amino acids that tend to accumulate under eT and eT+D conditions in comparison to Ctr and D regimes, respectively (**Figure 4C**). Proline and BCAAs which accumulated primarily under eC, eC+D and eC+eT+D regimes have been reported to have osmoregulatory roles (Girija et al., 2022). On the other hand, eC alleviated the effect of eT on the accumulation of amino acids, as the accumulation of Tyr and Lys decreased under eC+eT and eC+eT+D regimes when compared to *aC* counterparts (**Figure 4C**).

The metabolite profiling identified 10 phenolic compounds significantly (*P* ≤ 0.05) accumulated among the treatments (**Supplementary Table 2**). The scores plot between the first and second components identified two distinct groups related to the *aT* and *eT* regimes (**Figure 4A**). Based on the PLS-DA and VIP scores, 3hyd, Feru, and Epgg were identified as the key metabolites varying under treatment conditions (**Figure 4B**). The accumulation of these phenolics was temperature-dependent and unlike amino acids, CO_2_ exposure did not affect their abundance (**Figure 4C**). Compared with eT and eC, the effect of D on metabolome was minor or negligible.

One-way ANOVA revealed that all the Krebs cycle-related intermediates were significantly (*P* ≤ 0.05) accumulated among the climate variable treatments (**Supplementary Table 2**). Similarly, to the phenolic compounds, the scores plot between the first and second components identified two distinct groups associated with the *aT* and *eT* regimes (**Figure 4A**). Acids, such as α-ketoglutaric (Ket), Pyru and malic acid (Mal), were discriminated from other Krebs cycle intermediates based on the PLS-DA and VIP score (**Figure 4B**). An eT treatment increased contents of Pyru and Ket, while the content of Mal decreased (**Figure 4C**).

Fructose (Fruc), glucose (Gluc), sucrose (Suc), and galactinol (Galc) were the most significantly (*P* ≤ 0.05) accumulated saccharides among the climate variable treatments (**Supplementary Table 2**). The pattern of saccharide abundance showed two distinct groups associated with the *aC* and *eC* regimes (**Figure 4A**). Based on the PLS-DA and VIP score (> 1.0), Fruc and Gluc were identified as the most important saccharides whose abundance was mainly associated with the concentration of CO_2_ (**Figures 4B, C**). Apart from the above metabolic compounds, nitrogenous bases such as uracil (U), guanine (G) and thymidine monophosphate (TMP) were found to be significantly (*P* ≤ 0.05) accumulated (**Supplementary Table 2**). The most accumulated fatty acids at different regimes were identified to be linoleic acid (Lino), oleic acid (Olei), and palmitoleic acid (Palm) (**Supplementary Table 2**).

The correlation between the 44 significant metabolites and physiological traits was also evaluated. The analysis identified a significant negative correlation between the physiological traits (*Gsw* and *Tr*) and the accumulation of Fruc, Gluc, Arg, and shikimic acid (Shi) (**Supplementary Table 3**). Contrastingly, WUE showed positive correlation with these compounds.

## Discussion

Changing environment causes profound physiological and metabolic changes in the plants (Gianoli and Molina-Montenegro, 2021; Zandalinas et al., 2022). While the recent years have witnessed substantial advances in understanding the acclimation responses of wheat genotypes to various environmental drivers, most of the studies so far have mainly focused on single- or two-factor treatments, with limited possibility to assess the impacts of their mutual interactions (Zandalinas et al., 2018). Hence, the question of how plants will acclimate to a comprehensive future change in growth conditions is still far from complete. Therefore, understanding the underlying molecular mechanisms of adaptation response is crucial not only for the basic understanding of wheat stress response but also for applied wheat breeding. Through quantification of key metabolites, we reveal a differential metabolomic response against eight climate regimes (single and combined treatment) comprised of increased temperature, drought, and elevated CO_2_ in spring wheat.

The general mechanism to cope with changes in the environment involves an adjustment in the physiological and molecular traits (Pandey et al., 2017; Sharma et al., 2021; Alamri et al., 2022; Sharma et al., 2022). The shift in physiological responses can be attributed to specific abiotic factors and their nature i.e., individual and combined (Zandalinas and Mittler, 2022). Gas exchange measurements suggested that at the single-factor level, the effect of eC was predominant and led to the significant changes in *Tr*, *Gsw* and WUE, while eT and D had only minor effects. Similar patterns of physiological traits were also observed under double-factor regimes combining elevated CO_2_ with eT or D. As no prominent effect of eT and D on *Tr* and *Gsw* under aC was observed, this indicated that the response of these traits was mainly controlled by CO_2_ enrichment either at higher temperatures or decreased soil water availability. However, the physiological pattern exhibited in single and double factor combinations inclusive of an eC regime (eC+D and eC+eT) were not replicated in the triple factor regime (eC+eT+D). This suggests that the effect of eC could have been compromised by the simultaneous counteraction of the other two factors (eT and D). Higher *A* and *Tr* were observed during eC+eT+D treatment. In general, eC and eT have opposing effects on *Tr* (Kirschbaum and McMillan, 2018). An increase in the temperature enhances the *Tr*, however, due to partial stomatal closure under high CO_2_ concentration environment, a reduction in the *Tr* could be expected (Kirschbaum and McMillan, 2018). The increase in *Tr* under eC+eT+D in our study could be due to the influence of D, which could have helped to curtail the damage to the photosynthesis process (indicated by high *A*) through an evaporative cooling mechanism (Šigut et al., 2015).

The next focus was on investigating the metabolite accumulation to understand their role in plant acclimation under various regimes. The metabolic change patterns were highly treatment specific. For example, eC dominantly controlled the accumulation of soluble monosaccharides (Gluc and Fruc). A concomitant increase in the photosynthetic activity (*A*) was also observed in eC and the triple factor combinations eC+eT+D (**Figure 2D**). As monosaccharides are critical molecules for the primary carbon metabolism and the first substrates for starch biosynthesis, these results indicate that the accumulation of Gluc corresponded to the supplementary carbon fixation in these samples. The effect of higher CO_2_ concentration on the accumulation of compounds related to the sugar metabolites has also been reported in Arabidopsis thaliana (Ainsworth and Rogers, 2007; Zinta et al., 2018), and crops like wheat (Levine et al., 2008) and rice (Ainsworth, 2008). Moreover, saccharides are well known to regulate the rate of transpiration, particularly *via* stomatal dynamics (Li et al., 2018). The significant correlation identified in this study between the Gluc and Fruc accumulation with *Tr* and WUE of wheat leaves under various regimes could be attributed to the stomatal function.

On the other hand, metabolites associated with the Krebs cycle and phenolics were found to be mostly dependent on the exposure of temperature (**Figure 4**). High-temperature stress has been found to affect the levels of pyruvic acid, fumarate, malate, and citrate in poplar and soybean (Sicher, 2013; Ren et al., 2019). The spring wheat may have an inherent capacity to withstand the projected temperature increase (4°C) in the future climate by limiting the conversion of soluble sugars to organic acids of the Krebs cycle and secondary metabolites during elevated temperatures. The increase of carbon flux under eC conditions may then be utilized for synthesizing the compounds needed for acclimation responses and defense mechanisms as has been reported previously (Kiani-Pouya et al., 2017). Moreover, at the molecular level, the expression of genes linked with saccharides, Krebs cycle intermediates, and phenolics changes dynamically upon exposure to climate change-related factors (Krasensky and Jonak, 2012; Glaubitz et al., 2015; Xalxo et al., 2020). This may consequently facilitate modifications in the levels of many saccharides and phenolic compounds imparting specific acclimation responses. Accumulation of Feru (important for cell wall rigidity and strength), 3hyd (potential antioxidant), and Epgg (flavonoid) was dominantly controlled by the high temperature (**Figure 4**), suggesting that these compounds may help to maintain the cellular integrity and functionality during interaction with the environment.

Amino acids are an important molecular form of organic nitrogen in plants acting as intermediates for many metabolic reactions (Trovato et al., 2021). The pattern of amino acid accumulation exhibited a unique trend where eT played a pivotal role in the accumulation of amino acids like Tyr and Lys, but their enhanced accumulation pattern diminished when combined with eCO_2_ (**Figure 4**). CO_2_ enrichment has been previously shown to reverse the effects of elevated growth temperatures on many soluble amino acids in soybean leaves (Sicher, 2013). Noticeably, the level of amino acid and Krebs cycle intermediates showed contrasting patterns in eT and eC regimes, suggesting that a subtle balance may exist between these pathways.

In general, phenolics play a pivotal role in plants as antioxidants to scavenge the ROS upon exposure to abiotic stresses (Rice-Evans et al., 1997). The increase in their cellular concentration suggests the activation of redox homeostasis to protect against the possible damage to proteins and lipids (Awasthi et al., 2015). Similar to our study, an increase in the production of phenolics, and particularly flavonoids, under heat stress has been reported previously (Wu et al., 2016). Overall, these results show that the differential accumulation of metabolites regulates plastic response under future climate scenarios.

Our study represents a comprehensive analysis of metabolite homeostasis under different climate scenarios. This study highlighted that the metabolomic acclimation of spring wheat to a single and combination of climate factors is mainly associated with temperature and CO_2_ concentration. An environment with elevated CO_2_ mitigated some of the effects of elevated temperature on physiological performance and metabolism possibly by maintaining the rate of carbon fixation and accumulation of key metabolites and intermediates linked with sugar metabolism, Krebs cycle, and synthesis of phenolics. Usually, drought stress is manifested by substantial changes in plant metabolome including changes in simple saccharides that have a role of osmolytes. However, in this study, the effect of D on physiology and metabolome was minor/negligible. It is possible that the drought exposure applied to the targetted stage (Feekes-6) was likely mild, or for a short duration. In conclusion, the key metabolites and pathways identified in this study and their interactions with climate variables will be helpful in understanding their future climate-induced regulation and the manipulations of potential targets for the development of climate resilient and nutritionally enriched wheat crops (Irfan and Datta, 2017). Further, a better understanding of these strategies can provide an impetus to research on gene function discovery and biochemical evolution, which is foundational for improved metabolic engineering.

## Data availability statement

The original contributions presented in the study are included in the article/**Supplementary Material**. Further inquiries can be directed to the corresponding author.

## Author contributions

PPS and KK conceptualized the research. KV, SP, MO, HF, BV, EO, KKO-A, and PPS were involved in the methodology and analysis. SP, KV, PPS, OU and KK wrote and edited the manuscript. All authors have read and agreed to the final version of the manuscript.

## Funding

This research was funded by Czech Science Foundation, grant no. 20-25845Y (awarded to PPS). A part of the research was also supported by the project SustES “Adaptation strategies for sustainable ecosystem services and food security under adverse environmental conditions” (CZ.02.1.01/0.0/0.0/16_019/0000797) to SP, BV, OU and KK. Infrastructure facilities used in the experiments were supported by the Ministry of Education, Youth and Sports of CR within the CzeCOS program, grant number LM2018123HF.

## Acknowledgments

We express our gratitude to the Prof. RNDr. Ing. Michal V. Marek, Director of the Global Change Research Institute CAS, Brno for providing access to the necessary facilities and support.

## Conflict of interest

The authors declare that the research was conducted in the absence of any commercial or financial relationships that could be construed as a potential conflict of interest.

## Publisher’s note

All claims expressed in this article are solely those of the authors and do not necessarily represent those of their affiliated organizations, or those of the publisher, the editors and the reviewers. Any product that may be evaluated in this article, or claim that may be made by its manufacturer, is not guaranteed or endorsed by the publisher.
