# Supplementary material for: Single and interactive effects of variables associated with climate change on wheat metabolome
Source: Front Plant Sci. 2022 Oct 10;13:1002561. doi: 10.3389/fpls.2022.1002561 (PMC9589161; doi:10.3389/fpls.2022.1002561)
Supplement: Supplementary file 2 [file Table_2.docx]

| **Supplementary Table 2.** Important metabolite identified by one-way ANOVA and post-hoc analysis of the leaf metabolites in cv. Cadenza at vegetative stage. | | | | | | |
| --- | --- | --- | --- | --- | --- | --- |
| **Metabolites** | **Code** | **Categories** | **f.value** | **p.value** | **-LOG10(p)** | **FDR** |
| Lysine | Lys | Amino Acids | 43.06 | 3.30E-09 | 8.4815 | 8.25E-08 |
| Tyrosine | Tyr | Amino Acids | 29.158 | 5.86E-08 | 7.2322 | 6.28E-07 |
| Serine | Ser | Amino Acids | 26.11 | 1.30E-07 | 6.886 | 1.17E-06 |
| Methionine | Met | Amino Acids | 25.831 | 1.40E-07 | 6.8525 | 1.17E-06 |
| Glycine | Gly | Amino Acids | 23.875 | 2.46E-07 | 6.6083 | 1.54E-06 |
| Hydroxy-proline | Hpro | Amino Acids | 16.138 | 3.72E-06 | 5.4292 | 1.74E-05 |
| Threonine | Thr | Amino Acids | 15.733 | 4.42E-06 | 5.355 | 1.95E-05 |
| Phenylalanine | Phe | Amino Acids | 13.846 | 1.03E-05 | 4.9868 | 3.87E-05 |
| Leucine | Leu | Amino Acids | 11.062 | 4.35E-05 | 4.3617 | 0.000155 |
| Proline | Pro | Amino Acids | 10.005 | 8.10E-05 | 4.0917 | 0.000264 |
| Histidine | His | Amino Acids | 7.8859 | 0.000331 | 3.4798 | 0.000887 |
| Asparagine | Asp | Amino Acids | 7.0031 | 0.000645 | 3.1903 | 0.001613 |
| Glutamine | Glu | Amino Acids | 6.744 | 0.000793 | 3.1007 | 0.001879 |
| Tryptophan | Try | Amino Acids | 6.7304 | 0.000802 | 3.0959 | 0.001879 |
| Glutamic acid | Gln | Amino Acids | 3.4816 | 0.018379 | 1.7357 | 0.03362 |
| Arginine | Arg | Amino Acids | 6.2875 | 0.001156 | 2.9372 | 0.002626 |
| Linoleic acid | lino | Fatty Acids | 4.71 | 0.004911 | 2.3088 | 0.009444 |
| Oleic acid | olei | Fatty Acids | 4.2839 | 0.007597 | 2.1194 | 0.014244 |
| Hexadecenoic acid | 6deco | Fatty Acids | 3.433 | 0.019444 | 1.7112 | 0.034721 |
| Lactic acid | Lac | Kreb Cycle Acids | 18.271 | 1.60E-06 | 5.7959 | 8.57E-06 |
| Pyruvic acid | Pyru | Kreb Cycle Acids | 24.094 | 2.31E-07 | 6.6365 | 1.54E-06 |
| Oxalacetic acid | Oxa | Kreb Cycle Acids | 17.924 | 1.82E-06 | 5.7388 | 9.12E-06 |
| L-(-)-Malic acid | Mal | Kreb Cycle Acids | 10.731 | 5.26E-05 | 4.2794 | 0.000179 |
| ?-Ketoglutaric acid | Ket | Kreb Cycle Acids | 9.084 | 0.000145 | 3.8387 | 0.000418 |
| Succinic acid | Suc | Kreb Cycle Acids | 7.4748 | 0.000449 | 3.3479 | 0.001161 |
| Shikimic acid | Shi | Kreb Cycle Acids | 5.8734 | 0.001651 | 2.7823 | 0.003641 |
| Citric acid | Cit | Kreb Cycle Acids | 5.7102 | 0.001908 | 2.7194 | 0.004088 |
| Guanosine | G | Nitrogenous bases | 50.365 | 1.02E-09 | 8.9933 | 3.81E-08 |
| Uracil | U | Nitrogenous bases | 39.003 | 6.91E-09 | 8.1608 | 1.29E-07 |
| Thymidine monophosphate | TMP | Nitrogenous bases | 9.4241 | 0.000116 | 3.9342 | 0.000349 |
| Ferulic acid | Feru | Phenolics | 169.76 | 8.37E-14 | 13.077 | 6.28E-12 |
| Gallic acid | Gall | Phenolics | 34.09 | 1.87E-08 | 7.7283 | 2.34E-07 |
| Protocatechuic acid | cate | Phenolics | 18.354 | 1.55E-06 | 5.8095 | 8.57E-06 |
| Epigallocatechin | Epig | Phenolics | 14.955 | 6.20E-06 | 5.2078 | 2.58E-05 |
| 3-hydroxybenzoic acid | 3hyd | Phenolics | 14.804 | 6.63E-06 | 5.1786 | 2.62E-05 |
| Epigallocatechin gallate | Epgg | Phenolics | 9.6117 | 0.000103 | 3.9859 | 0.000323 |
| Vanillic acid | Vani | Phenolics | 8.081 | 0.000288 | 3.5408 | 0.0008 |
| Quercetin | Quer | Phenolics | 5.6526 | 0.002009 | 2.697 | 0.004186 |
| Caffeic acid | Caff | Phenolics | 3.3493 | 0.021439 | 1.6688 | 0.037393 |
| Luteolin | Lute | Phenolics | 3.2083 | 0.025333 | 1.5963 | 0.043181 |
| Fructose | Fruc | Saccharides | 37.291 | 9.64E-09 | 8.0161 | 1.45E-07 |
| Glucose | Gluc | Saccharides | 25.075 | 1.74E-07 | 6.7602 | 1.30E-06 |
| Sucrose | Succ | Saccharides | 5.5854 | 0.002135 | 2.6706 | 0.004328 |
| Galactinol | Galc | Saccharides | 5.5353 | 0.002235 | 2.6508 | 0.00441 |
