# Supplementary material for: Single and interactive effects of variables associated with climate change on wheat metabolome
Source: Front Plant Sci. 2022 Oct 10;13:1002561. doi: 10.3389/fpls.2022.1002561 (PMC9589161; doi:10.3389/fpls.2022.1002561)
Supplement: Supplementary file 3 [file Table_3.docx]

**Supplementary Table 3**: Pearson's correlation value (r) between the physiological traits and metabolites. The significant associations (P < 0.05) are highlighted in bold.

| **Label** | **A** | **gsw** | **Tr** | **WUE** |  |
| --- | --- | --- | --- | --- | --- |
| U | 0.02 | -0.33 | -0.51 | 0.49 |  |
| G | 0.07 | 0.21 | 0.26 | -0.32 |  |
| TMP | -0.51 | 0.24 | 0.34 | -0.46 |  |
| 3hyd | -0.07 | -0.15 | 0.13 | -0.23 |  |
| cate | 0.18 | -0.35 | -0.25 | 0.37 |  |
| Vani | 0.23 | 0.21 | 0.12 | 0.05 |  |
| Gall | -0.30 | 0.16 | 0.21 | -0.34 |  |
| Caff | -0.46 | -0.29 | -0.46 | 0.36 |  |
| Feru | -0.08 | -0.11 | 0.09 | -0.24 |  |
| Lute | 0.12 | -0.70 | **-0.80** | **0.88** |  |
| Quer | 0.56 | 0.46 | 0.39 | -0.25 |  |
| epig | -0.58 | 0.04 | -0.06 | -0.18 |  |
| epgg | 0.00 | 0.17 | 0.37 | -0.42 |  |
| Ket | -0.40 | -0.05 | 0.16 | -0.28 |  |
| Pyru | -0.02 | -0.34 | -0.06 | -0.04 |  |
| Shi | -0.22 | **-0.87** | **-0.92** | **0.95** |  |
| Cit | -0.39 | 0.22 | 0.06 | -0.13 |  |
| Mal | 0.49 | 0.25 | 0.02 | 0.23 |  |
| Suc | 0.30 | 0.28 | 0.01 | 0.12 |  |
| Oxa | 0.14 | 0.64 | 0.63 | -0.59 |  |
| Lac | 0.22 | 0.50 | 0.39 | -0.27 |  |
| Gly | 0.00 | 0.61 | 0.68 | -0.67 |  |
| Leu | 0.54 | -0.55 | -0.40 | 0.50 |  |
| Ser | -0.22 | 0.55 | 0.68 | **-0.77** |  |
| Thr | -0.56 | -0.21 | -0.28 | 0.12 |  |
| Met | 0.20 | **-0.72** | -0.68 | 0.67 |  |
| Lys | 0.34 | -0.50 | -0.32 | 0.34 |  |
| asp | -0.41 | -0.35 | -0.37 | 0.38 |  |
| Glu | -0.03 | 0.64 | 0.66 | -0.69 |  |
| gln | -0.44 | -0.64 | -0.53 | 0.30 |  |
| Arg | 0.03 | **-0.86** | **-0.80** | **0.80** |  |
| His | 0.03 | **-0.77** | -0.59 | 0.54 |  |
| Phe | 0.42 | -0.29 | -0.08 | 0.18 |  |
| Try | -0.12 | 0.40 | 0.40 | -0.46 |  |
| Pro | -0.43 | 0.58 | 0.58 | -0.67 |  |
| Tyr | -0.04 | -0.41 | -0.13 | 0.07 |  |
| Hpro | -0.14 | 0.00 | -0.05 | 0.20 |  |
| 6deco | -0.14 | 0.55 | 0.56 | -0.66 |  |
| lino | 0.01 | 0.36 | 0.40 | -0.43 |  |
| olei | -0.03 | 0.61 | 0.51 | -0.52 |  |
| fruc | 0.18 | **-0.85** | **-0.79** | **0.82** |  |
| gluc | 0.23 | **-0.78** | **-0.75** | **0.80** |  |
| suc | -0.58 | 0.24 | 0.13 | -0.19 |  |
| galc | -0.20 | 0.48 | 0.31 | -0.37 |  |
